# A Novel Energy Planning Scheme Based on PGA Algorithm and Its Application

**DOI:** 10.1155/2022/1722848

**Published:** 2022-08-21

**Authors:** Xian-Long Lv, Shikai Tang, Jia Su

**Affiliations:** ^1^School of Electrical and Information Engineering, Tianjin University, Tianjin 300072, China; ^2^College of Electrical Engineering and Automation, Shandong University of Science and Technology, Qingdao 266590, Shandong, China; ^3^School of Electrical Engineering and Automation, Taiyuan University of Technology, Taiyuan, Shanxi, China

## Abstract

In order to actively respond to the “14th Five-Year Plan,” the PGA algorithm is used to develop a new energy planning strategy in this paper. The project can make full use of my country's abundant renewable energy resources, encourage energy conservation and reduction of emissions, improve the energy structure's low-carbon level, support the development of smart green energy, and achieve ecological civilization construction. This solution can show users how much greenhouse gas emissions can be reduced through some environmental changes, as well as the basic issues of meeting the future energy needs. It can display the benefits, costs, and emissions data under different scenarios in the future and use the scenario demonstration method to show energy planning to make energy data more vivid. It allows people, technicians, and decision makers to understand what will happen to China's carbon emissions over time in the next 15 years. This paper innovatively combines a particle swarm optimization algorithm with a genetic algorithm and designs a PGA algorithm for path optimization. In terms of carbon emission reduction, comparative trials demonstrate that the PGA algorithm's path optimization is 58.06 percent greater than the genetic algorithm; In terms of cost, the PGA algorithm's path optimization is 15.72% less expensive than the genetic algorithm's. This article provides a reference path for selecting the best results for future energy planning schemes and provides a new strategy for the “14th Five-Year” energy plan.

## 1. Introduction

Although my country is relatively rich in fossil energy, renewable energy, and hydropower, most of the energy development is difficult, costly, and technically demanding. The demand for various energy sources is increasing as my country's economy develops rapidly. According to statistics this year my country's energy output has shown a downward trend, and energy distribution in some regions is severely uneven and divergent. This has caused large-scale, long-distance, and high-cost energy transportation such as transmission of West-East Gas and transmission of West-East Power, which has caused energy problems to continue to increase. Articles [1] describe the energy plan will reduce environmental pollution, improve energy efficiency, increase GDP, and a series of advantages. The research results of the article [2–4] can conclude that energy planning plays a major role in promoting my country's national economy and social developmzent. Energy planning is an overall arrangement for the structure, development, production, conversion, use, and distribution of energy based on the prediction of the corresponding energy demand according to the national economic and social development plan of a certain period. Therefore, the current energy planning, energy innovation, and energy security establishment have become the focus of my country's development.

My country's power consumption is expanding in tandem with its rapid economic expansion, and the problem of power shortages remains difficult to fully resolve. State Grid's long-term development strategy includes the creation of “a worldwide leading energy Internet enterprise with Chinese features.” This goal will further promote the development process of grid energy interconnection and sharing and mutual benefit, and it is also a process of using Internet technology to transform and upgrade the traditional power grid. It has put forward the objective of constructing a modern socialist country in all aspects, as well as the vision and principles of attaining “green” and sustainable development, to advance the 14th Five-Year Plan and the 2035 long-term goal. The creation of a low-carbon economy is at the heart of my country's strategy to attain this aim. The low-carbon economy is a sustainable economic development model advocated to deal with climate change, according to the article[5]. Articles [6, 7] studies have shown a significant role in a low-carbon economy for the sustainable development of our country played.

Although energy planning is a relatively new field in my country, it can alleviate my country's energy problems to a certain extent. So far, many projects related to energy planning have entered the implementation stage in various directions [8]. For example, the Energy Institute of Beijing Tsinghua Urban Planning and Design Institute proposed a new urban energy planning method that is more suitable for Chinese cities [9], can effectively solve the contradiction between rapid urban development and energy shortage, synchronize the urbanization process and the efficient use of scarce energy supplies; the London 2014 Summer Olympics will use the 2006 building code as the baseline, and by 2013, it will improve the heat, power, and cooling system. The energy conversion and transmission efficiency of the power supply system and the installation of renewable energy power generation systems to achieve the goal of 50% reduction in CO2 emissions have achieved 47% in actual conditions, but they have also achieved good results.

Since large-scale construction and infrastructure projects require long-term planning, it takes a long time for energy planning to be realized. As a result, there will be a lot of unknowns in the process of planning for the long-term target of 2035. For example, it is impossible to accurately predict which low-carbon technologies will flourish and which will fail, what the low-carbon power generation mix will look like, or how behavior and infrastructure will change or need to change during the intervention period. A more accurate prediction of the direction of energy development can enable the masses, staff, and the government to plan the future energy development more clearly and accurately and provide accurate energy planning plans for the future.

Although regional energy planning can guarantee the sustainable development of the energy industry, there are still some difficulties in operation, development, and technology. Difficulties in energy planning and operation mainly include: not many targets for energy conservation and emission reduction; not many that have a return on investment and economic benefits; not many that achieve multi-energy integration; some economic and energy benefits are not ideal; the instability of energy prices and energy policies. Difficulties in the development of energy planning mainly include: the scale of development is small and most areas are not implemented. In terms of energy planning technology, it mainly includes: no standards and specifications; energy planning is not linked to demand planning, but rather to supply planning; benefit appeals bundle technology and the deviation of different professional understanding of technology.

The above-mentioned problems have led to the slow progress of my country's energy planning and the unsatisfactory results. This work presents a new energy planning system based on the PGA algorithm to address some of the issues mentioned above, which can make various energy data vivid; it can show users to what extent greenhouse gas emissions can be reduced through certain environmental changes and the basic problem of meeting energy demand in the future. It shows the benefits, costs, and emissions data of different scenarios in the future, allowing the users to plan some changes that will occur in various directions in the next 15 years to provide a reference for the current development path. To accurately predict the energy trend, this planning scheme uses the elastic coefficient method prediction model and the regression analysis method prediction model. The elasticity coefficient method is the ratio of the growth rates of two connected economic indicators over time. It is a measure of the relationship between the growth rates of two economic variables and is frequently used to anticipate energy supply and demand income and expenditure. Regression analysis refers to an analysis method that uses the principles of data statistics to process a large amount of data and find a certain rule from it, establish a correlation function and extrapolate it to predict future changes. It is mainly used in market supply and demand, economic analysis, and input and output.

Although forecasting future scenarios is challenging, a successful transition to a low-carbon economy needs a clear goal and early action. The proposed energy planning scheme can solve the problems of fewer emission reduction targets, unstable return on investment, and integration of multiple energy sources. Realize the supply and demand requirements of diversified energy, realize a variety of combined paths while meeting the emission reduction targets and the requirements of energy supply and demand, and can choose the optimal path under a specific situation, providing users with diversified choices. And the applicability of this program is wide, and it can be applied to multiple regions.

Genetic algorithms and particle swarm algorithms are two algorithms that are often used in energy planning projects [10–12]. However, research has demonstrated that using a pure genetic algorithm or a particle swarm approach to determine the best solution for energy planning is problematic. Therefore, this paper draws on the research of other researchers [13–16], combines particle swarm algorithm and genetic algorithm, and proposes a new optimization algorithm called the PGA algorithm. Because both particle swarm algorithms and genetic algorithms are intelligent algorithms, they both initialize populations and particles randomly, and both have fitness values based on the optimization functions. The above conditions provide a basis for the combination of these two algorithms.

Genetic algorithms are derived from the theory of evolution and population genetics and are an important part of computational intelligence. Genetic algorithms are currently employed in a variety of domains, including scheduling issues [17], neural networks [18], engineering design [19], function optimization [20], and combination optimization [21]. The social behavior of birds or fish schools inspired the particle swarm algorithm. At present, the PSO has been widely used in neural networks [22], function optimization [23], classifier design [24], etc.

The PGA method uses the particle swarm algorithm to run a small number of iterations before keeping the *n* particles with the best fitness value for genetic algorithm crossover and mutation operations. The initial particles for the cross-mutation procedure are all derived from a short number of iterations of the particle swarm method, and the termination condition is then determined. The best result will be output if the conditions are met; otherwise, the particle velocity and position attributes will be updated again. Continue to iterate until the termination condition is met. After many tests, the results show that the path optimization of the PGA algorithm designed in this paper can increase the carbon emission reduction rate by up to 58.06% compared with the path optimization of the genetic algorithm. In terms of cost, the path optimization of the PGA algorithm can reduce up to 15.72% compared with the path optimization of the genetic algorithm. The program through the use of the PGA algorithm obtains the optimal results under different circumstances, such as the least expensive, least emission reduction, energy-efficient, and the highest level of electrification and other energy planning. These findings can be used as a starting point for energy planning programs.

The model of this energy scheme is briefly introduced in Section 1; the system structure of the energy scheme is introduced in Section 2; in Section 3, the PGA algorithm is introduced; the optimization results are shown in Section 4; and in Section 5, the article is summarized.

## 2. System Structure

### 2.1. Brief Description of the Model

This energy planning program is being developed in tandem with future energy development directions in order to accomplish the aim of a modern energy system that is “green, low-carbon, safe, and efficient.” Innovatively put forward a new energy planning method, under the premise of realizing the future energy development goals, clarifying the subgoals that need to be met in each energy field. Different development paths under the energy diversified development goal can be found. And the energy planning scheme combines the PGA algorithm to screen out the optimal results under different development goals.

The energy planning scheme optimization is split into two parts: input and output. Oil, natural gas, coal, power generation, electric power, and other inputs are included, and population, economy, industry, transportation, life, etc., are the output part. A total of 32 parameters describe the energy planning scheme. By looking for a certain correlation between the large amount of data collected over many years, and based on the understanding of the current situation, the continuous analysis of 32 kinds of data can finally predict the data to 2035 to realize the future scenario planning. This solution can answer the extent to which the users can meet the diversified needs of energy. This article also provides a preset optimization plan according to the user's conventional needs and provides a reference path for future energy planning, to select the best planning plan. Energy planning programs are tools to help decision makers, the energy industry, and the public understand these choices. This solution allows users to develop their own desired scenario path, and can independently design the input level of each parameter. By 2035, the target is to cut the greenhouse gas emissions by 80% from 2010 levels while ensuring that energy supply matches the demand.

#### 2.1.1. Analytical Method

Scenario analysis is a method often used in energy planning. According to the article [25], scenario analysis, which is based on the analysis of important unknown aspects in the future, can explain a variety of future scenarios and give support for customized high-quality energy development plans and policies. Considering that there will be some uncertainties in planning future energy scenarios this time, the scenario analysis method [25] is used in this plan to illustrate the potential results under alternative assumptions. The planning scheme model can be made as flexible as feasible by using this basic way to examine the potential path until 2035.

**Figure 1:**
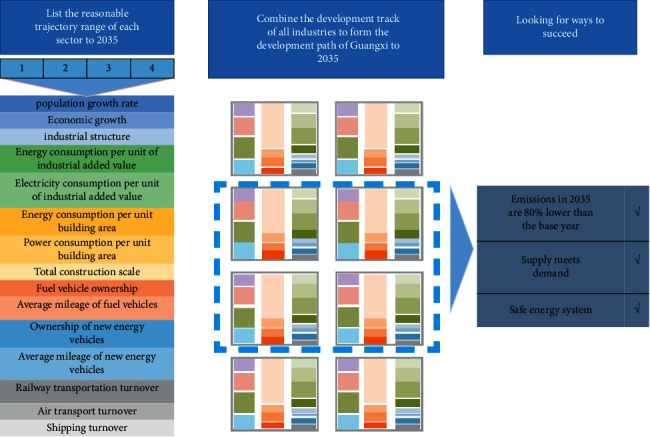
Energy planning process.

This program takes a sector-by-sector approach to better understand the magnitude and type of changes that may occur in each field of the emission and energy systems, so that the future scenarios can be modelled more precisely. For each industry sector, this article sets four different development trajectories that may exist in the next fifteen years. These trajectories contain all the potential possibilities of this industry. After understanding the trajectory range of each field, this article sets up a model for energy planning. The model can integrate multiple sectors' sectoral trajectories in various ways to determine all conceivable pathways in the next 15 years, from 2020 to 2035. This plan is not only able to see the plan for 2035, but also a series of changes that will occur every 5 years in the past 15 years, and they will be displayed vividly.  Figure 1 describes the process carried out.

#### 2.1.2. Energy Sector Trajectory

**Figure 2:**
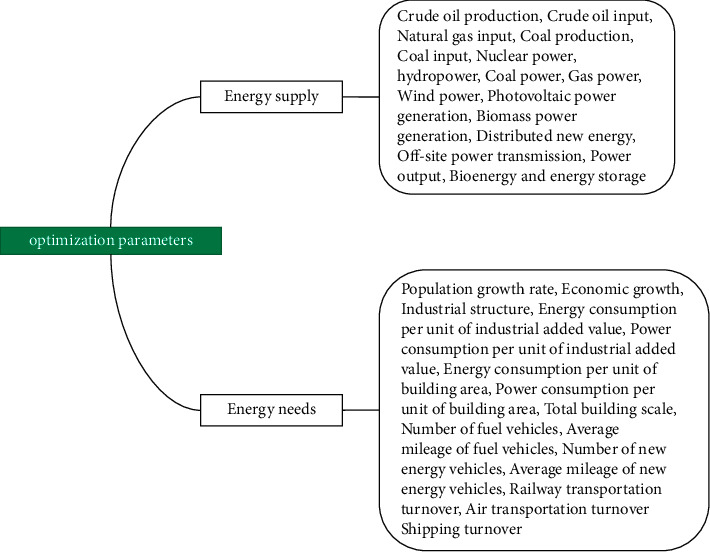
Optimization parameters.

Figure 2 lists the optimum input parameters, which are grouped into two categories: energy supply and energy demand.

**Figure 3:**
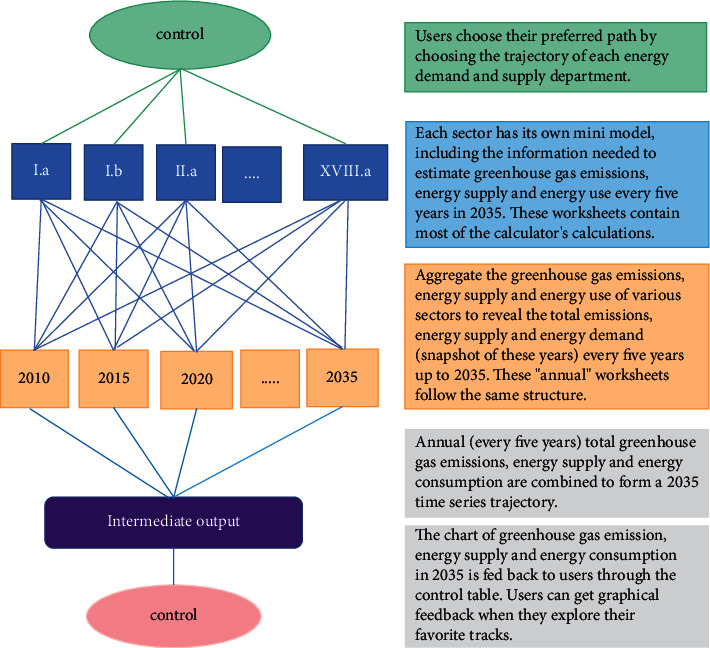
Trajectory of emissions, energy supply, and energy demand generated by this scheme.

Figure 3 depicts how Guangxi's 2035 energy planning strategy generates emissions, energy supply, and energy demand:

In this plan, the energy trajectory of different energy production industries is investigated, in view of the types of possible changes in each industry, the plan designs four development directions from low to high. This can be thought of as the industry's four potential levels of economic development over the next 15 years, representing continuous efforts to change in the next 15 years. The purpose is to cover the wide range of possibilities in each department and test the range of possible existence. This form can be observed in all potential situations that may be experienced in the industry. The four potential levels are shown as follows:

  Direction 1: assume that this direction is that no decarbonization technology is implemented, or only a small part of it is implemented, there is no low-carbon technology development or deployment.
  Direction 2: assume that this direction is to achieve some possible goals in a reasonable way. For some departments, this will be similar to the successful completion of the currently ongoing program or the expected construction effect of the ongoing project.
  Direction 3: assume that this direction is a goal that can only be achieved through a very large level of effort. If there are no major changes in current technology, this direction is impossible to achieve; it assumes a major breakthrough on the technical level.
  Direction 4: assuming that this direction is a goal that can be achieved through extreme efforts, this level will reach the physical or technical limitations.

The demand department explored several different drivers of change.

Changes in residents' behavior and lifestyle: reducing food waste; accepting lower average room temperature in winter; and changing transportation methods, such as changing from private transportation to public transportation, to reduce energy demand and emissions.

Technology development and improvement: green low-carbon technologies, such as LED lighting and ground source heat pumps, are being developed and used; existing technologies, such as new industrial technologies, are being improved; and current technologies are being made more efficient. The deployment of technologies that are still being developed will determine the development trajectory.

Different technologies or fuel choices: the level of technology is not directly related to the choice of fuel, such as the choice between district heating or ground source heat pumps, or the choice between automobile fuel cells and new energy batteries. Try to choose energy with fewer carbon emissions as possible.

Structural changes: reflect possible changes in the economic structure and compare with emission targets and energy supply requirements.

#### 2.1.3. System Structure

**Figure 4:**
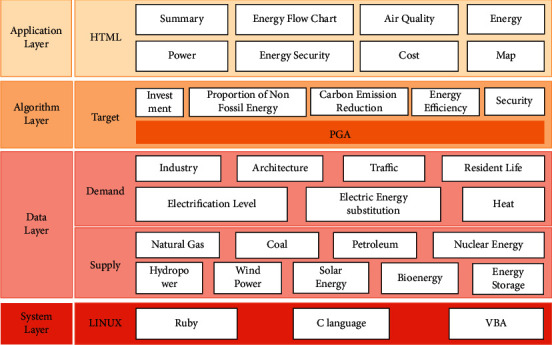
System architecture diagram.

The system consists of four levels, which are application level, algorithm level, data level, and system level. The system architecture diagram is shown in Figure 4.

The application layer is mainly to display the interface output results including an overview, energy flow diagram, air quality, energy, electricity, energy security, cost, and map. It visually shows the development and changes of each output over time.

The algorithm layer applies the PGA algorithm to optimize the desired goals of the system, including investment, nonfossil energy share, carbon emission reduction, energy efficiency, and safety.

The data layer contains the various data required by the system, which is mainly divided into two categories: demand and supply. Demands include industry, construction, transportation, residents' lives, electrification level, electric energy substitution, and heat. Supplies include natural gas, coal, petroleum, nuclear power, hydropower, wind power, solar power, biomass, and energy storage. These data are predicted to 2035 for each category. Each data is divided into four grades. Choosing a different grade will affect the results of the plan.

The system layer is the underlying code using Ruby language, C language, and VBA language to achieve.

## 3. PGA Algorithm Model and Application

**Figure 5:**
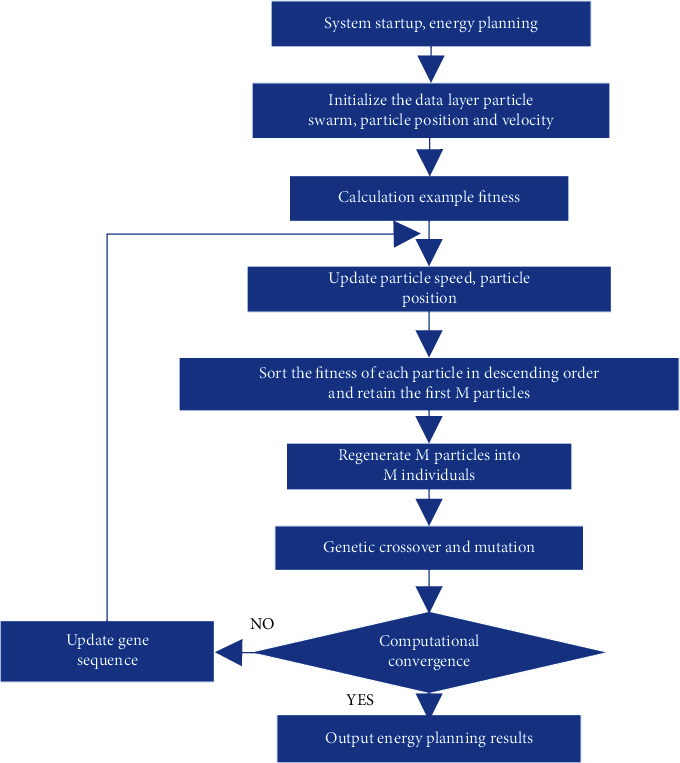
Flowchart of PGA algorithm in the energy planning scheme.

The model flowchart of the PGA algorithm in this scheme is shown in Figure 5. Optimization targets include carbon emission reduction, energy investment, energy efficiency, safety, and the proportion of nonfossil energy sources. All optimized routes are not the optimum routes since there are too many unknown elements. These illustrative paths, on the other hand, aid in the selection of the best future option.

### 3.1. Generating Initial Particle Population

First, initialize the particle swarm randomly, the number of particles is 2000, and the dimension is 53, of which 21 genes are used as extensions and the value is set to 0, and the remaining 32 are divided into four levels (gene values are 1 to 4). The particle position and velocity are shown in the following formula (1), and then the fitness of these particles is calculated.

(1) $$\begin{array}{c} {\overset{-g}{X}}_{i}=\left\{x_{i1}^{g},x_{i2}^{g}\ldots x_{i53}^{g}\right\},\\ {\overset{-g}{V}}_{i}=\left\{v_{i1}^{g},v_{i2}^{g}\ldots v_{i53}^{g}\right\}, \end{array}$$

where *X*_*ij*_^*g*^ is particle position, the size is 53, and *V*_*ij*_^*g*^ is particle velocity, the size is 53.

### 3.2. PSO Optimization GA Selection Crossover Mutation

Secondly, perform 40 iterations of the PSO algorithm for the random initial population. The first 1000 with the best fitness continue to execute the selection cross mutation of the GA algorithm, and the remaining particles are retained. The new particles generated by the GA are re-added to the previously retained particles and then enter the next iteration of the combined algorithm.

The particle group velocity update equation is shown in the following formula:

(2) $$\begin{array}{c} v_{\text{id}}^{k+1}=x\cdot \left[v_{\text{id}}^{k}+c_{1}\times {\text{rand}}_{1}\times \left(p_{\text{id}}^{k}-x_{\text{id}}^{k}\right)+c_{2}\times {\text{rand}}_{2}\times \left(p_{g\;d}^{k}-x_{\text{id}}^{k}\right)\right], \end{array}$$

where *x* is the inertia weight; *c*_1_ and *c*_2_ are different learning factors; rand_1_ and rand_2_ are random numbers distributed between 0 and 1; and *x*_id_^*k*^ is the current position of the particle; *p*_id_^*k*^ is the optimal solution found by the particle in the *k*th theory iteration, which is the individual extreme value; *p*_*gd*_^*k*^ is the optimal solution found by the entire population in the *k*th theoretical iteration, that is, the global extremum.

The updated particle swarm position formula is shown in the following formula:

(3) $$\begin{array}{c} x_{\text{id}}^{k+1}=x_{\text{id}}+v_{\text{id}}^{k+1}, \end{array}$$

where *x*_id_^*k*^ is the position of the *k*th generation of the *d*th particle.

The selection operation embodies the principle of survival of the fittest, selecting high-quality individuals through the degree of adaptation, and weeding out low-quality individuals. The selection method used in this article is the ranking selection method among the 23 selection methods summarized in the article published [26, 27] by Potts et al. Crossover is a way to form a new individual; it is to exchange part of genes in some way between two chromosomes that cross each other. The crossover method used in this article is the uniform crossover among the 17 crossover methods summarized by Potts et al. The mutation operation refers to replacing some values in the individual code string with other genes to form a new individual.

After the PSO algorithm is optimized, the first 1000 excellent particles are obtained, and then the crossover and mutation in the GA algorithm are performed on the first 1000 particles. Crossover refers to randomly generating individuals with serial numbers from 1 to 1000, denoted as father and mother. The probability that the new individual generated comes from the father and mother is the same, both are 50%. Variation refers to the nth dimension of the 53 returned by detecting the individual sequence. If not 0, a number between 0 and 5300 will be produced at random. If the created number equals 0, redefine the nth number, generate a random number between 1 and 4, and create a new individual. After that, the regenerated 1000 individuals are combined with the previously retained particles and enter the next iteration of the combined algorithm again.

### 3.3. Outputting the Best Result

Finally, after generating the initial population, the optimization process is iterated for 40 generations. In the process, new individuals are constantly being created. Finally, the system outputs the best sorted result.

### 3.4. Experimental Results and Analysis

To verify that the optimization speed and optimization accuracy of the PGA algorithm proposed in this paper is better than the genetic algorithm when solving the optimization problems. In this paper, under the condition that the operating environment of the two algorithms of PGA and GA remains unchanged, eight examples are selected from the low-dimensional and high-dimensional perspectives to test the algorithm. The 8 test functions are shown in Table 1. These 8 functions are all minimization problems. Among them, the low-dimensional functions are *f*1–*f*4, and 4 general standard test functions are used, namely, the traditional function Griewank function, the rotation function Rotated Griewank function, the transfer function Shifted Griewank function, and the complex function Shifted rotated Griewank function. The maximum number of calls for each algorithm is 3.00*E* + 05. The high-dimensional function is *f*5–*f*8, using four of the 20 1000-dimensional test functions announced by the CEC2010 conference, namely, the Separable function, the Single-group m-nonseparable function, the *D*/2*m*-group *m*-nonseparable function, and the *D*/*m*-group m-nonseparable function. The maximum number of calls for each algorithm is 3.00*E* + 06.

The parameters are set as follows: the population size is set to 50, the maximum number of iterations is 100, the learning factors *c*1, *c*2 = 3, the cross mutation probability pc = 0.35, pm = 0.1, and the similarity threshold is 0.01.

The experimental results are shown in Table 2.

The optimization effect of the method is judged in the low-dimensional test function by the three features of solving mean value, solving ratio, and solving speed, as shown in Table 2. The findings of the PGA method suggested in this research are clearly superior to the GA algorithm in these four test circumstances. Especially in function *f*2, the GA algorithm cannot solve the result, while PGA can solve the result of this function.

As shown in Table 3, in the high-dimensional test function, the optimization effect of the algorithm is judged from the global optimal solution and the number of calculations. It can be seen that the global optimal solution of the PGA algorithm proposed in this paper in these four high-dimensional functions is the closest to the theoretical value, and the number of calculations is less. Most of the results of the GA algorithm in these four high-dimensional functions are far from the theoretical value and the calculation times are many.

Combining two tables can be drawn by PGA proposed algorithm which is in all respects superior to the ordinary GA algorithm.

## 4. Optimization Results

### 4.1. Optimization Results of the PGA Algorithm

By using the PGA algorithm and the GA algorithm, respectively, several different paths of carbon emission reduction and cost results were optimized. They are: No changes in all aspects, highest demand without supply, highest supply without demand, lowest cost, highest proportion of nonfossil energy, highest proportion of nuclear energy, highest proportion of fossil energy, highest carbon emission reduction, highest energy efficiency, highest level of electrification, highest carbon emission reduction, highest energy efficiency, highest level of electrification, highest carbon emission reduction, highest energy efficiency, highest level of electrification development that lasts. These different paths are designed to take into account the economic development, energy consumption and some extreme cases, covering all economic possibilities. These paths provide guidance for different directions of energy planning.

The results of the PGA algorithm optimization path are compared with the GA algorithm optimization path results as shown in Table 4 (the data in parentheses are the GA algorithm optimization results). Comparing the two data, it can be seen that the PGA algorithm proposed in this paper is better than the optimization result of the GA algorithm.

The results of using the PGA algorithm to optimize the demand, supply, and emissions of each path in 2035 are shown in Table 5.

Air Pollution Health Impact Index: this index measures the impact of air pollutants including SO2, NOx, NMVOC, BC (black carbon), CO, and primary PM2.5 on human health. According to the research of article [28] and article [29], it is found that air pollutants have various effects on human health, and the risk of health effects such as mild symptoms of illness or death will increase with the increase of pollutant concentration. Using 2010 as the base year, figures below 100 indicate a decrease in average air pollution and its associated health effects, while figures above 100 show an increase (100). The index measures change in the province's average concentration of air pollutants but do not reveal the number or severity of pollution hotspots [30, 31].

Table 6, which is divided into the worst case and best case scenarios of air pollution, examines the impact of each path on the air quality in 2035 in 2010. The worst case scenario for air pollution is that, aside from the current strategy, between now and 2035, there will be no additional deployment or innovation to reduce emissions. The best situation for air pollution is that effective technologies to improve or eliminate environmental pollution can be developed between now and 2035 and have been fully used.

Table 6 shows the impact of each path on air quality in 2010. We can do a good job in energy planning according to the content in the table, according to their own actual situation, planned to develop effective technology to improve or eliminate environmental pollution, and give full play to the guiding significance of energy planning.

## 5. Conclusion

This article provides a plan to explore a series of potential energy planning paths from today to 2035, and consider some of the difficult choices and trade-offs we will have to make. The study drew some common elements of feasible approaches and explored the implications and uncertainties associated with different options. It exposes the magnitude and speed of the required changes, as well as some of the major decisions that will need to be made. Based on previous research, a new type of PGA algorithm is proposed. Through this algorithm, other paths under different conditions are optimized, which provides a reference path for future planning. After testing, the PGA algorithm designed in this paper is superior to the genetic algorithm in various aspects. In this solution, the path optimization of the PGA algorithm in the carbon emission reduction ratio can increase by up to 58.06% compared to the genetic algorithm path optimization; in terms of cost, the path optimization of the PGA algorithm can reduce up to 15.72% compared with the genetic algorithm path optimization. On the basis of this research, an energy planning scheme suitable for other regions and even the whole country can be developed. With the development of our country, the data on energy is constantly updated, and this plan also needs to be continuously optimized with the development of our country, so that this plan is more realistic, convenient for users to operate, and more vivid to show the planning results.

## Figures and Tables

**Table 1:** Test case.

Serial number	Test function	Theoretical value
*f*1	$f_{1}=1+\left(1/4000\right)\sum_{i=1}^{n}x_{i}^{2}-\prod_{i=1}^{n}\text{cos}\left(x_{i}/\sqrt{i}\right)\left[-500,500\right]$	0
*f*2	*f*_2_=*f*_1_(*y*_*i*_), [−500,500]	0
*f*3	*f*_3_=*f*_1_(*z*_*i*_)+*f*_bias1_, *f*_bias1_=−180[−500,500]	−180
*f*4	*f*_4_=*f*_1_(*z*_*i*_)+*f*_bias2_*z*_*i*_=(*x*_*i*_ − *o*) × *M* *f*_bias2_=−180, [−500,500]	−180
*f*5	*f*_5_=∑_*i*=1_^*n*^(10^6^)^(*i* − 1/*n*−1)^*z*_*i*_^2^, [−100,100]	0
*f*6	*f*_6_=*f*_rot_elliptic_[*z*(*P*_1_ : *P*_*m*_)] × 10^6^+*f*_elliptic_[*z*(*P*_*m*+1_ : *P*_*n*_)], [−100,100]	0
*f*7	*f*_7_=∑_*k*=1_^(*n*/2*m*)^*f*_rot_elliptic_[*z*(*P*_(*k* − 1)×*m*+1_ : *P*_*k*×*m*_)]+*f*_elliptic_[*z*(*P*_(*n*/2)+1_ : *P*_*n*_)], [−100,100]	0
*f*8	*f*_8_=∑_*k*=1_^(*n*/*m*)^*f*_rot_elliptic_[*z*(*P*_(*k* − 1)×*m*+1_ : *P*_*k*×*m*_)][−100,100]	0

**Table 2:** Results of 25 experiments on low-dimensional test cases.

		GA	PGA
*f*1	Mean	2.58*E* − 10	0.00*E* + 00
	Solving rate	100.00%	100.00%
	Speed	3.40*E* + 05 s	6.23*E* + 03 s

*f*2	Mean	1.64*E* + 00	0.00*E* + 00
	Solving rate	0.00%	100.00%
	Speed	Inf	7.41*E* + 03 s

*f*3	Mean	6.47*E* − 08	0.00*E* + 00
	Solving rate	100.00%	100.00%
	Speed	9.46*E* + 04 s	1.98*E* + 04 s

*f*4	Mean	3.33*E* − 02	0.00*E* + 00
	Solving rate	56.67%	100.00%
	Speed	5.18*E* + 05 s	1.55*E* + 04 s

**Table 3:** Results of 25 experiments on high-dimensional test cases.

		GA	PGA
*f*5	Global optimal solution	6.48*E* − 04	4.66*E* − 04
	The average number of calculations	28900	17800

*f*6	Global optimal solution	1.86*E* + 13	3.11*E* + 01
	The average number of calculations	30000	16200

*f*7	Global optimal solution	4.21*E* + 06	6.74*E* − 04
	The average number of calculations	30000	25350

*f*8	Global optimal solution	1.02*E* + 09	4.99*E* − 04
	The average number of calculations	30000	24300

**Table 4:** PGA algorithm vs. GA algorithm with the same number of iterations.

Path name	Carbon emissions (PGA, GA)	Cost (yuan/person/year) (PGA, GA)
No changes in all aspects	6% (4%)	4115 (4775)
Maximum demand, no supply	65% (47%)	4301 (4886)
Maximum supply, no demand	49% (31%)	5874 (6203)
Lowest cost	73% (55%)	3810 (4521)
Nonfossil energy has the highest proportion	79% (62%)	4512 (5037)
Nuclear energy accounts for the highest proportion	83% (65%)	4703 (5324)
Fossil energy accounted for the highest proportion	80% (63%)	4640 (5253)
Highest carbon reduction	96% (78%)	4891 (5402)
Highest energy efficiency	81% (62%)	4143 (4804)
Highest level of electrification	84% (66%)	4537 (5124)
Sustainable development	80% (61%)	4011 (4639)

**Table 5:** Supply and demand emissions results.

Path name	Demand (TWh/yr)	Supply (TWh/yr)	Emission (10^4^*t*CO_2_/yr)
No changes in all aspects	1024	1137	20554
Maximum demand, no supply	356	517	7653
Maximum supply, no demand	885	2824	11152
Lowest cost	460	917	5903
Nonfossil energy has the highest proportion	463	558	4592
Nuclear energy accounts for the highest proportion	690	1129	3717
Fossil energy accounted for the highest proportion	569	732	4373
Highest carbon reduction	440	581	875
Highest energy efficiency	532	607	4155
Highest level of electrification	601	790	3499
Sustainable development	484	603	4373

**Table 6:** Comparison of the impact of each path on air quality in 2035 compared to 2010.

Path name	The worst case of air pollution	Best case for air pollution
No changes in all aspects	68	26
Maximum demand, no supply	24	9
Maximum supply, no demand	55	20
Lowest cost	43	14
Nonfossil energy has the highest proportion	33	13
Nuclear energy accounts for the highest proportion	43	15
Fossil energy accounted for the highest proportion	65	13
Highest carbon reduction	23	8
Highest energy efficiency	31	10
Highest level of electrification	34	13
Sustainable development	33	12

## Data Availability

The datasets generated during and/or analyzed during the current study are not publicly available due to sensitivity and data use agreement.

## References

[1] Bi T., Ju M., He Y., Shao C. An overview of my country’s energy planning and its environmental impact assessment theory.

[2] Xu W., Sun Z., Feng X., Du G., Li J. (2013). Development status and prospects of regional energy technology. *Building Science*.

[3] Zhu Y. (2000). The enlightenment of medium and long-term energy development scenario analysis method to my country’s future energy saving planning. *China Energy*.

[4] Wang L. (2006). Discussion on the main points of China’s energy strategy and planning. *Science and Technology Information Development and Economy*.

[5] Bao J., Yang M., Chen F. (2008). Low-carbon economy: a new change in human economic development. *China Industrial Economy*.

[6] Yang L. (2010). My country’s low-carbon economic development path selection and policy recommendations. *Urban Development Research*.

[7] Jia D. Low-carbon Economy and Industrial Structure Adjustment in China.

[8] Shi X. (2007). Several renewable energy plan projects of PetroChina have entered the implementation stage. *Geothermal Energy*.

[9] Fu L., Zheng Z., Jiang Y. (2008). Urban energy planning method based on dynamic and spatial distribution. *Urban Development Research*.

[10] Shuai X., Wang X., Li K., Wang B., Yuan S., Zhang L. Research on day-ahead dispatch of electricity-heat integrated energy system based on improved PSO algorithm.

[11] Brouwer A. S., van den Broek M., Seebregts A., Faaij A. (2014). Impacts of large-scale Intermittent Renewable Energy Sources on electricity systems, and how these can be modeled. *Renewable and Sustainable Energy Reviews*.

[12] May G., Stahl B., Taisch M., Prabhu V. (2015). Multi-objective genetic algorithm for energy-efficient job shop scheduling. *International Journal of Production Research*.

[13] Amjadi M. H., Nezamabadi-pour H., Farsangi M. (2010). Estimation of electricity demand of Iran using two heuristic algorithms. *Energy Conversion and Management*.

[14] Zhang D. *The Improvement and Application of Genetic Algorithm and Particle Swarm Algorithm*.

[15] Sun F. J., Tian Y. Transmission Line Image Segmentation Based GA and PSO Hybrid Algorithm.

[16] Sang Y., Peng Z., Qian L. PSO-GA Collaborative Optimization Algorithm and its Application in Protein Structure Prediction.

[17] Valls V., Ballestín F., Quintanilla S. (2008). A hybrid genetic algorithm for the resource-constrained project scheduling problem. *European Journal of Operational Research*.

[18] Kharrati H., Khanmohammadi S., Pedrycz W., Alizadeh G. (2012). Improved polynomial fuzzy modeling and controller with stability analysis for nonlinear dynamical systems. *Mathematical Problems in Engineering*.

[19] Vural R. A., Yildirim T., Kadioglu T., Basargan A. (2012). Performance evaluation of evolutionary algorithms for optimal filter design. *IEEE Transactions on Evolutionary Computation*.

[20] Liu C., Hammad A., Itoh Y. (1997). Multiobjective optimization of bridge deck rehabilitation using a genetic algorithm. *Computer-Aided Civil and Infrastructure Engineering*.

[21] Hamid T., Osian S. H., Nafiseh S., Naghmeh S. Solving the Traveling Salesman Problem Using Genetic Algorithms with the New Evaluation Function.

[22] Tu J. (2013). *Research and Application of PSO Optimization Neural Network Algorithm*.

[23] Peng S., Wu Z. (2013). Research on Rosenbrock function optimization problem based on improved PSO algorithm. *Computer Science*.

[24] Yu B. Research on Neural Network Classifier Based on Improved Particle Swarm Algorithm.

[25] Zhu J., Liu X. M., Pang W. H. (2015). Scenario Analysis for the Energy Demand and Carbon Emissions in Low Carbon City. *Ecological Economy*.

[26] Li J. P., Balazs M. E., Parks G. T., Clarkson P. J. (2002). A species conserving genetic algorithm for multimodal function optimization. *Evolutionary Computation*.

[27] Liu C., Huang F., Yang Z. (2015). A Meta analysis on the associations between air pollution and respiratory mortality in China. *Chinese Journal of Epidemiology*.

[28] Wang Q., Li T. T., Chen C., Sun Q. H., Cui L. L., Xu D. Q. (2013). The PM2.5 characteristics of haze pollution and the related health impacts in China. *Zhonghua Yixue Zazhi*.

[29] Lu Q. (2015). Google’s solar energy solution. *Energy*.

[30] Wang S. (2009). Online carbon trace solution. *China Paper*.

[31] People’s Daily (2020). *Proposals of the Central Committee of the Communist Party of China on Formulating the Fourteenth Five-Year Plan for National Economic and Social Development and Long-Term Goals for 2035*.

